# The impact of physician education regarding the importance of providing complete clinical information on the request forms of thrombophilia-screen tests at Tygerberg hospital in South Africa

**DOI:** 10.1371/journal.pone.0235826

**Published:** 2020-08-06

**Authors:** Ibtisam Abdullah, Andrea D. Jafta, Zivanai C. Chapanduka

**Affiliations:** 1 National Health Laboratory Service Tygerberg Hospital and Division of Haematological Pathology, Faculty of Medicine and Health Sciences, Stellenbosch University, Cape Town, South Africa; 2 AMPATH Laboratories and Division of Haematological Pathology, Faculty of Medicine and Health Sciences, Stellenbosch University, Cape Town, South Africa; Maastricht University Medical Center, NETHERLANDS

## Abstract

**Background:**

Thrombophilia-screen tests are specialised haemostasis tests that are affected by numerous unique patient variables including the presence of acute thrombosis, the concomitant use of medication and patient demographics. Complete information on the request form is therefore crucial for the haematological pathologist to make patient-specific interpretation of patients’ results.

**Objectives:**

To assess the completeness of thrombophilia-screen test request forms and determine the impact of provision of incomplete information, on the interpretive comments generated by reporting haematological pathologists. To assess the impact of an educational session given to clinicians on the importance of providing all the relevant information on the request forms.

**Method:**

Two retrospective audits, each covering 3 months, were performed to evaluate the completeness of demographic and clinical information on thrombophilia-screen request forms and its impact on the quality of the interpretive comments before and after an educational intervention.

**Results:**

One hundred and seventy-one request forms were included in the first audit and 146 in the second audit. The first audit revealed that all 171 thrombophilia-screen request forms had complete patient demographic information but none had clinical information. Haematological pathologists only made generic comments which could not be applied to a specific patient. The second audit, conducted after a physician educational session, did not reveal any improvement in the clinical information provision by the test-ordering physicians. This was reportedly due to the lack of space on the request form. The interpretive comments therefore remained generic and not patient-specific.

**Conclusion:**

Physicians’ failure to provide relevant clinical information made it impossible for pathologists to make patient-specific interpretation of the results. A single physician education session did not change the practice, reportedly due to the inappropriate design of the test request form. Further studies are required to investigate the impact of an improved request form and the planned electronic test requesting.

## Introduction

Thrombophilia-screen tests are requested in a select group of patients presenting with venous thromboembolism (VTE) to predict the likelihood of recurrence of thrombosis and to decide on the duration of anticoagulation therapy. Anticoagulation treatment increases the risk of bleeding therefore careful assessment is needed before intense, extended or life-long treatment is instituted. On the opposite pole, insufficient therapy predisposes to recurrent thrombosis.[1] Anticoagulation therapy not only carries the risk of bleeding but also increases patient morbidity and health care costs. Anticoagulation therapeutic agents are influenced by multiple factors including patient genomics, the concomitant use of other therapeutic drugs and diet. Warfarin, the most commonly used anticoagulant drug, has a narrow therapeutic window and needs to be closely monitored through an International Normalized Ratio (INR) test to ensure efficacy and safety.[2,3]

Patient demographics including age and gender influence the normal reference ranges of various parameters included on the thrombophilia-screen. Timing of sample collection and immediate delivery to the laboratory are imperative for thrombophilia-screen tests. Other variables to consider include underlying infections, liver disease, renal dysfunction, obstetric history, the presence of acute thrombosis and additional concomitant medication.[4]

The lupus anticoagulants (LAC) is an *in vitro* phenomenon of prolonged phospholipid-dependent clotting time due to acquired autoantibodies. These autoantibodies target the phospholipid-binding proteins of the cell membrane resulting in a paradoxical risk of thrombosis *in vivo*.[5] LAC test constitutes an important modality of screening for the acquired form of thrombophilia and can be falsely positive during the acute phase of thrombosis, pregnancy, exposure to oral contraceptive (OC), hormone replacement therapy (HRT) and anticoagulant therapy.[5–7]

Levels of the natural anticoagulants, which form part of the thrombophilia-screen, including Protein S (PS), Protein C (PC), and Antithrombin (AT), can be falsely low during the acute phase of thrombosis. Exposure to OC, HRT and anticoagulant medications also impact the natural anticoagulants and should be taken into account when interpreting test results.[6,8] The screening for heritable thrombophilia includes molecular testing for Factor V Leiden (FVL, FVR506Q) and Prothrombin (FII, F2G20210A) gene mutations. Although these mutations are associated with unequivocally increased risk of venous thrombosis, confirmatory testing is only indicated in a select group of patients.[8] The molecular tests for these mutations (e.g. Polymerase Chain Reaction analysis for Factor V Leiden and Prothrombin gene mutation) are not sensitive to pre-analytical patient variables.[7]

Anticoagulation therapy can vary from a few months to lifelong therapy, depending on the patients’ clinical presentation taken in combination with thrombophilia-screen test results. Furthermore, the intensity of the anticoagulation varies depending on the patients’ unique attributes.[5,9] The provision of all relevant clinical information on the request forms is therefore important for accurate test result interpretation and minimises the costs related to repeat testing.[10]

Studies evaluating the adequacy of information provided on request forms revealed that the clinical information is frequently inadequate.[11–14]

Interventions are necessary to improve the provision of demographic and clinical data upon requesting thrombophilia screening tests. The intervention strategies include test-requesting clinician education, strict sample rejection policies, improving the request form design and using electronic laboratory test requesting systems.[10,15–17]

The aim of this study is to investigate the completeness of information provided on thrombophilia-screen request forms and to assess the impact of any incomplete information on the interpretive comments of test results. Furthermore, the impact of a single educational session for clinicians is determined by re-assessing the information provided on the request forms after the educational intervention. To our knowledge, this is the first study conducted in a coagulation laboratory in South Africa that focused on the impact of insufficient clinical data on the interpretative comments of thrombophilia-screens before and after the education of requesting physicians on the importance of providing all relevant information on the request forms.

## Methodology

The study was conducted at the National Health Laboratory Service (NHLS) Coagulation laboratory at Tygerberg Hospital (TBH), a 1 384 bed multidisciplinary tertiary care academic hospital in the Western Cape province of South Africa. TBH is the main teaching hospital for the University of Stellenbosch’s Faculty of Medicine and Health Sciences. The Coagulation laboratory renders services to TBH and non-Tygerberg hospitals (NTBH). NTBH are surrounding tertiary- and district-hospitals as well as out-patient clinics, which refer patients and/or samples for testing to TBH.

This was a quasi-experimental, pre- and post-intervention study in which two retrospective audits were conducted. The audits analysed the completeness of the information provided on thrombophilia-screen request forms received from TBH and NTBH. The initial audit assessed the completeness of the request forms over 3 months (01 July—30 September 2017). The second audit was performed one month after the educational session. It assessed the same information over 3 months (01 November 2017–31 January 2018). Only TBH physicians participated in the educational session, thus the NTBH arm was the control group.

The following exclusion criteria applied:

- Requests only for genetic assays for Factor V Leiden and the Prothrombin 20210A mutations.
- Request forms referred from outside the Western Cape Province.
- Request for thrombophilia screening in individuals below the age of 18-years.

More than 90% of the physicians responsible for ordering thrombophilia screens participated in the educational session which was carefully designed to deliver key messages regarding the reasons why the clinical and demographic information was required. The pre-analytical factors affecting the test results were explained. These factors included; the timing of the specimen drawing, the order of specimen tube drawing, the use of tourniquets, the impact of underfilling or overfilling of the specimen tubes, the specimen temperature and the time allowed between specimen draw and analysis. The effect of patient demographics and attributes such as the presence or absence of acute thrombosis at the time of testing, renal and liver disease, infection, pregnancy, and the concomitant use of anticoagulant and other medication, was explained. The impact of inadequate information on the pathologist’s ability to make a patient-specific interpretation of the results was emphasised. The educational session consisted of a 30 minute Microsoft PowerPoint® presentation by the principal investigator and 15 minutes of open discussion and questions.

Provision of the following parameters on the request form was assessed;

- Patient demographics: name, surname, folder number, date of birth and gender.
- Hospital, clinic and/or ward information.
- Requesting physician information: name, speciality and contact details (including official telephone numbers, pager numbers or cell phone numbers).
- Clinical information: presumptive clinical diagnosis, documented presence of acute thrombosis, anticoagulant therapy and other concomitant medications.
- Date and time of specimen collection.

The impact of incomplete clinical information was measured by assessing the interpretive comment provided for abnormal results. If a generalised or generic comment was provided, the impact was considered significant. A generic comment in this study was defined as any comment that could not be applied to the specific patient. (Table 4)

The impact of the education session was measured by comparing the information supplied on request forms referred from TBH, where the educational session was conducted, and NTBHs, where no educational session occurred.

Data was captured using Microsoft Excel^®^ software and the Statistical Package for the Social Sciences (SPSS) version 25 was used for data analysis. The Pearson’s chi-square test was used to compare categorical outcomes between sites, and between the pre- and post-intervention assessments at the same site. The odds of having complete data for each field on the form were estimated using multiple logistic regression analysis, with the site (TBH versus NTBH), time period (pre- versus post-educational intervention) and the interaction between site and timing to indicate an intervention effect.

### Ethical consideration

The study was approved by the Ethics Committee of the University of Stellenbosch (project registration number HEA-2017-1494) and was performed according to the Helsinki Declaration (2000) guidelines. The request forms were collected retrospectively and a waiver of consent was obtained. Data was collected by a single investigator to maintain consistency and confidentiality. The data capture sheet included the different variables and whether the forms had the complete information or not. Patient confidentiality was maintained and patient identifying information was not included on the data capture sheet on which patients were identified by a study number only.

## Results

Three hundred and sixty-six request forms were received (195 and 171 in the first and second audit periods respectively). Based on the exclusion criteria, 46 forms were excluded and 3 forms were not retrievable, leaving 317 forms (171 and 146 in the first audit and second audit periods respectively). In the first audit, 128 request forms were received from TBH and 43 from NTBH. In the second audit, 114 forms were from TBH and 32 were from NTBH.

**Fig 1:**
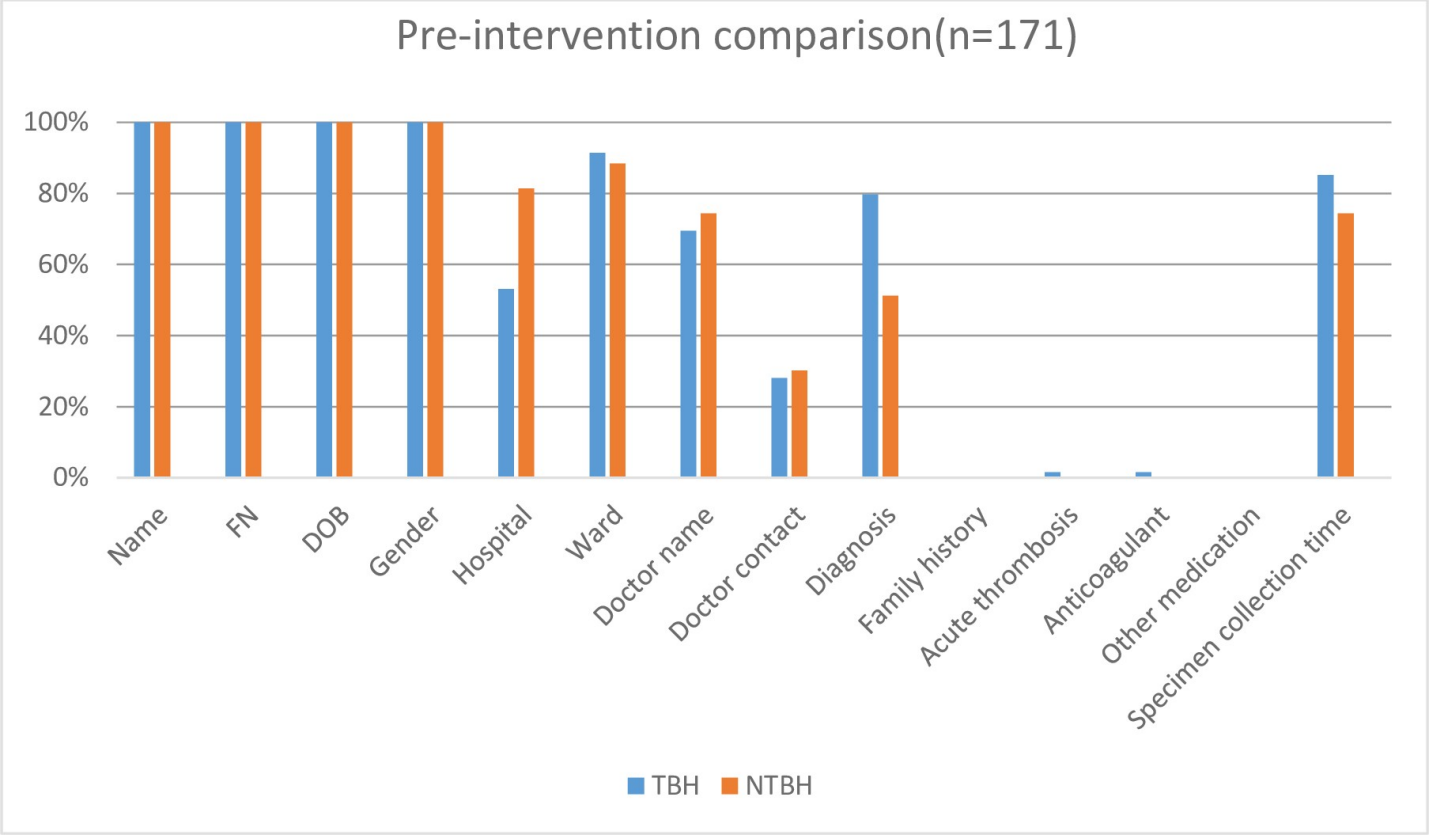
Pre-educational intervention percentage of complete data on the request forms: Tygerberg hospital versus non-Tygerberg hospitals. TBH–Tygerberg Hospital, NTBH–non-Tygerberg Hospital, FN—folder number, DOB—date of birth.

**Fig 2:**
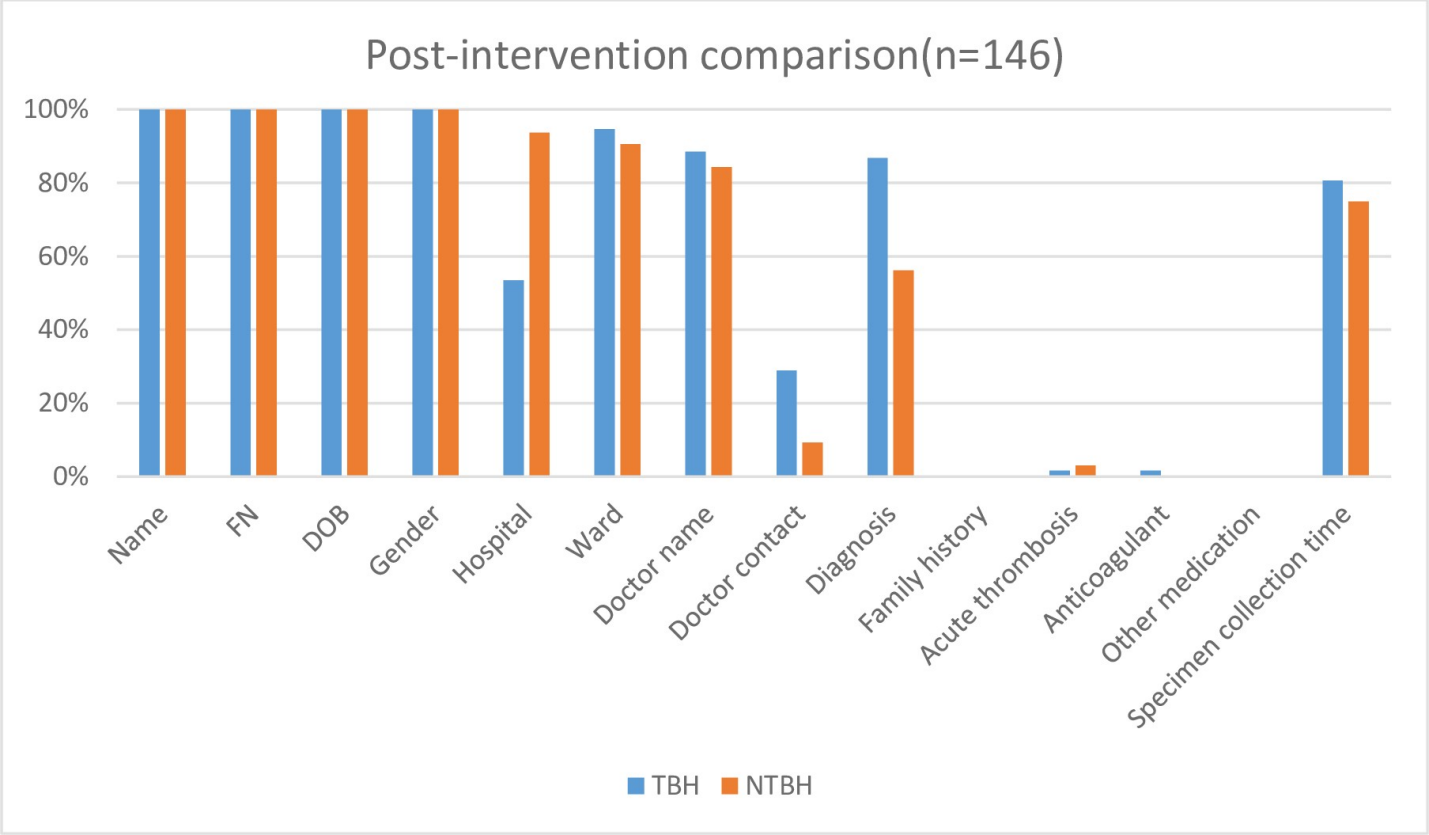
Post educational intervention percentage of complete data on the request forms: Tygerberg hospital versus non-Tygerberg hospitals. TBH–Tygerberg Hospital, NTBH–non-Tygerberg Hospital, FN—folder number, DOB—date of birth.

**Fig 3:**
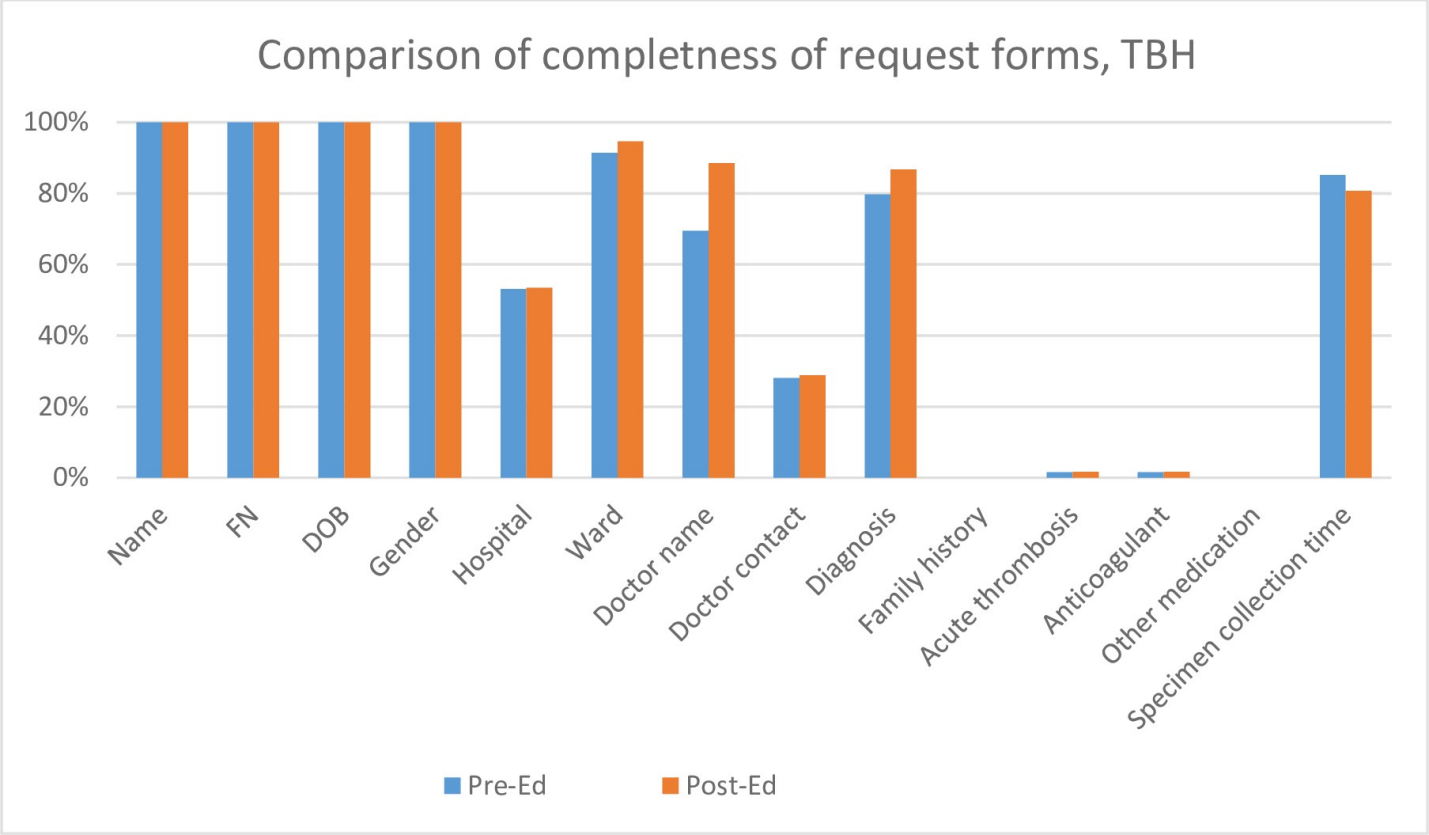
Comparison of the completeness of request forms referred from Tygerberg hospital (TBH) pre- and post-the educational session during the first and second audit. FN—folder number, DOB—date of birth, Pre-Ed—first audit results before the educational session at Tygerberg hospital, Post-Ed, second audit results after the educational session.

All forms from TBH and NTBH, pre- and post-educational intervention had patient demographics, which consisted of patient name, hospital folder number, date of birth and gender. This information was on the computer-generated sticker that was affixed to the request form. All request forms evaluated from TBH and NTBH, pre- and post-educational intervention had insufficient clinical information. (Figs 1–3, Tables 1 and 2).

**Table 1:** Comparison between Tygerberg hospital and non-Tygerberg hospital practice of filling request forms pre- and post-educational intervention.

Parameter	First audit (pre-education)			Second audit (post-education)		
	Incomplete forms TBH (%)	Incomplete forms NTBH (%)	p-value	Incomplete forms TBH (%)	Incomplete forms NTBH (%)	p-value
FH of VTE	100	100		99	100	
Thrombosis/Medication	98	100		98	97	
Diagnosis	80	51	p<0.01	87	56	p<0.001
Contact details	28	30	p = 0.7	29	9	p<0.001

TBH–Tygerberg Hospital, NTBH–non-Tygerberg Hospital, FH—Family history, VTE—Venous thrombo-embolism, Thrombosis/Medication—presence of acute thrombosis and anticoagulant medication, Contact details—contact details of the requesting doctor.

**Table 2:** Comparison of the completeness of request forms from Tygerberg hospital pre- and post the educational intervention.

Parameters	Pre-intervention (%)	Post-intervention (%)	p-value
Patient demographics	100	100	
Requesting physician name	70	89	p<0.001
Contact details	28	29	p = 0.888
Diagnosis	80	87	p = 0.139
Clinical information	98	98	
The time of specimen collection	85	81	p = 0.356

Contact details—contact details of the requesting doctor.

The odds of completion of select variables in the post-education and control groups was compared using logistic regression analysis. There was no significant improvement in the intervention group in comparison to the control group, as shown by the non-statistically significant interaction term in the models below (Table 3). The exception was in the case of doctor contact details which showed statistically significant (p<0.05) improvement in inclusion in the post-education group as compared to the control group (Table 3). The odds of improving the completion of doctor contact details on the forms from the intervention group were 0.055 over time, regardless of the intervention. Request forms from the intervention group were 0.2 times more likely to have the doctor contact details than those from the control group, regardless of time or intervention (Table 3).

**Table 3:** Logistic regression analysis of the impact of the educational intervention.

		p-value	OR	95% CI for OR	
				Lower	Upper
Provision of ward	Timing	0.978	0.956	0.039	23.260
	The interaction between intervention and control groups regardless of time	0.970	1.052	0.073	15.091
	Interaction	0.759	1.331	0.214	8.275
	Constant	0.030	5.712		
Provision of diagnosis	Timing	0.912	0.895	0.125	6.389
	The interaction between intervention and control groups regardless of time	0.252	2.732	0.490	15.238
	Interaction	0.591	1.371	0.433	4.335
	Constant	0.592	0.764		
Provision of doctor contact details	Timing	0.040	0.055	0.003	0.871
	The interaction between intervention and control groups regardless of time	0.116	0.207	0.029	1.479
	Interaction	0.049	4.361	1.007	18.890
	Constant	0.001	0.099		

OR—Odds Ratio, CI—Confidence Interval, Timing—The effect of time on intervention and control groups regardless of the education, Interaction—The effect of intervention on intervention and control groups considering time pre- and post-intervention

In the first audit, 38 (22%) and in the second audit 23 (16%) thrombophilia screens had abnormal test results. The interpretative comments provided by the haematopathologists were not customised for the specific patient but were generic. Examples of the generic comments that were provided are shown on Table 4. A conclusive diagnosis was not made and only lists of differential diagnoses were provided.

**Table 4:** Examples of the generic comments that were provided by pathologists.

Abnormal tests	Generic comment
Protein C	Protein C deficiency can be inherited or acquired. Acquired protein C deficiency is noted in patients on OC / HRT, Vit K antagonists, Vit K deficiency, DIC, liver disease and acute thrombosis. Please correlate clinically.
Protein S	Protein S deficiency can be inherited or acquired. Acquired causes include normal pregnancy, OC/HRT, Vit K antagonists, DIC, liver disease and antiphospholipid syndrome. Please correlate clinically.
Antithrombin	Antithrombin deficiency can be heritable or acquired. Acquired causes include pregnancy, OC, heparin therapy, asparaginase therapy, DIC, current massive thrombosis, severe liver disease, nephrotic syndrome and inflammatory bowel disease. Please correlate clinically.

OC–Oral contraceptive, HRT–Hormonal replacement therapy, DIC–Disseminated intravascular coagulation.

Relevant clinical information including the presence of acute thrombosis at the time of specimen collection, family history of thrombosis, use of anticoagulation therapy or other medications at the time of testing was incomplete in almost all (98.4%) request forms pre- and post-intervention. The lack of inclusion of this information on the request forms rendered it impossible to assess the odds ratio and the p-value in these variables.

All respondents in a sample of the post-education session TBH doctors revealed that the reason for not providing clinical information on the request form was that there was insufficient space on the form to enter the required information.

## Discussion

The pre-analytical phase in laboratory testing includes procedures that are out of the control of the laboratory personal such as completion of request forms.[18] Several studies conducted to evaluate the completeness of request forms, have highlighted the risk of insufficiently completed forms.[12,13,19] Few studies have examined the exact frequency and clinical impact of incomplete laboratory request forms.[11,19] Previous studies highlighted that the request for the appropriate laboratory tests and provision of the relevant clinical information on the request forms are important for a quality laboratory service and reduction of unnecessary costs.[10,11,20]

This study found that the patient demographic data was the best-provided data on the request forms referred for thrombophilia-screen tests, which was in agreement with other studies.[11,12,19–21] The satisfactory completion of demographic data on all the request forms assessed, is largely due to the patient-specific, electronically generated stickers which are mandatory patient identifiers.

The essential clinical information necessary for appropriate interpretation of thrombophilia-screen test results was lacking on all request forms. This is consistent with many studies which established that clinical information is one of the least completed sections on the laboratory request form.[11–14] A South African study conducted by Zemlin *et al* focused on the impact of incomplete information on laboratory request forms with regards to interpretive comments from pathologists. The authors concluded that the lack of clinical data can lead to an inappropriate assessment of thyroid function test results.[19]

A similar previous study indicated that insufficient data on the laboratory request forms influenced the interpretive comment.[21] In this study, clinical information including the diagnosis or presumptive diagnosis, the presence or absence of acute thrombosis at the time of sample collection, family history of thrombosis, past thrombotic episodes, patient exposure to anticoagulation therapy as well as other medications taken at the time of sample collection such as oral contraceptives, was inadequate on all request forms evaluated. The presence of acute thrombosis decreases PC, PS and AT levels, which can result in a false impression of inherited deficiency. Acute thrombosis can also give rise to false-positive LAC test results. Thrombophilia-screen tests collected at the time of an acute thrombotic episode or while the patient is on anticoagulant therapy can, therefore, be unreliable.[4]

Interpretation of thrombophilia-screen results takes into account the provisional diagnosis; the time elapsed after the thrombotic event, exposure to medication and patient age.[15] In this study, the absence of the relevant clinical data made it impossible for pathologists to provide an interpretive comment. Only generalised generic interpretive comments, which could have not applied to the specific patient, were possible. Other factors could be postulated to have caused the pathologists to make generic comments. However, clearly, in the absence of the necessary information, it would not have been possible to make patient-specific comments.

Omission of test-requesting physician details impairs communication of critical laboratory results to the treating physician or ward staff and make it impossible for laboratory staff to request any further patient-specific information.[11] In the first audit, contact details of the requesting doctor, were absent on 122 (70.8%) request forms of which 50 (38.8%) lacked both a legible doctor’s name and contact details.

Burnett L *et al*. showed that manually completed requesting doctor details can lead to insufficient, incorrect or illegible data on request forms and that the use of ink-stamps personalised with the name, unique doctor registration number and contact details improved the availability of this information on request forms.[10]

The time of specimen collection is important in coagulation-based thrombophilia-screen test analysis and analysis should ideally be performed within 4 hours of blood collection. In the first audit, the time of specimen collection was not stated in 30 (17.5%) request forms. These requests should ideally be rejected. Due to the magnitude of the problem, it was decided to accept the requests pending the systematic collection of data from this study. A comment pointing out the reasons for the need to state the collection time and the impact of its omission was made on the reports.

Studies have been performed to analyse the impact of remedial interventions on the adequate completion of laboratory request forms by clinicians.[10,15–17] I. Osegbe *et al*. evaluated the effectiveness of clinician education on improving the practice of completing request forms. The study showed improvement after clinician education.[22] However, in our study education alone failed to resolve the problem. The lack of improvement in providing clinical information is most probably due to the inappropriate design of the current request form (S1 Appendix). This was stated in a sample of TBH physicians who were quizzed as to why they did not improve their practice despite the education. Studies have evaluated the effectiveness of different types of request forms in improving the completeness of data provided by clinicians.[10,15] Bailey *et al* showed that a redesigned request form reduced the frequency of requesting inappropriate tests.[17]

A study conducted by Majed Al Dogether *et al*. evaluated a manual compared to an electronic request form and concluded that the latter improved the quality of the information provided on the request forms.[16] Electronic-based laboratory request forms are not available at TBH.

Based on the finding of this study we recommend a combination of regular educational events about the importance of proper completion of request forms and the implications of incomplete request forms in addition to other remedial measures. It is recommended that a new method of capturing the required information be designed. This should include a new format for the current request form, a separate request form for thrombophilia screening and electronic ordering.

## Study limitations

### The small sample size

There was no formal question to establish the reason why pathologists only made generalised, generic interpretive comments due to the authors’ knowledge that it was impossible for patient-specific comments to be made without the relevant clinical information.

## Conclusions

The lack of relevant patient clinical information made it impossible for pathologists to provide clinically useful patient-specific interpretive comments on thrombophilia screening tests. A single educational event failed to improve the provision of such information and this was attributed to the poor design of the thrombophilia screening test request form (S1 Appendix). Recommended interventions include the use of a newly designed request form (S2 Appendix), electronic test ordering and an ongoing programme of physician education. Further studies will be performed to assess the effectiveness of the redesigned form.

## Supporting information

S1 Data (XLSX)

S1 Appendix (PDF)

S2 Appendix (PDF)
