# Supplementary material for: The impact of physician education regarding the importance of providing complete clinical information on the request forms of thrombophilia-screen tests at Tygerberg hospital in South Africa
Source: PLoS One. 2020 Aug 6;15(8):e0235826. doi: 10.1371/journal.pone.0235826 (PMC7410402; doi:10.1371/journal.pone.0235826)
Supplement: S1 Appendix — (PDF) [file pone.0235826.s002.pdf]

[illegible]

# CLINICAL PATHOLOGY

FOR LAB LABELS

## PLEASE PRINT IN BLOCKS AND ( X ) THE APPLICABLE BOXES

**MARK IF URGENT** ☐

**Patient I.D. Number**

### LOCATION

HOSPITAL / CLINIC

Ward

Cost Centre

### PATIENT INFORMATION

Patient Hosp./ Clinic Number

Fee class

Surname

First name

Address

PATIENT LABEL

Postal Code

Tel/Cell

Sex M F

D.O.B. D D M M Y Y Y Y

Age

### CLINICAL / SPECIMEN DETAILS

Diagnosis / reason for request

Medication

Warf

Hep

Type of specimen

Taken on at h

Taken by

### PRIVATE PATIENTS ONLY

ICD10 diagnosis codes:

Medical Aid:

Plan:

Medical Aid No:

Employer:

Authorisation No:

Dep code:

Account to / Principal Member:

Member address:

Postal Code

Member Tel. No. (H/Cell):

(W):

Member I.D. number:

### CONTACT DETAILS OF RESPONSIBLE PRACTITIONER

NAME ( Prof / Dr / Sr )

Persal or Practice No

Cell No

Bleep

Tel(0 )

Fax (0 )

Signature #

### CHEMICAL PATHOLOGY

| | | Rands |
|---|---|---|
| **General:** | | |
| HS* | Blood gases | 45 |
| Y | Sodium | 25 |
| Y | Potassium | 25 |
| Y | Chloride | 15 |
| Y | Urea | 25 |
| Y | Creatinine | 25 |
| Y | Calcium | 25 |
| Y | Magnesium | 25 |
| Y | Inorganic phosph | 25 |
| Y | Uric acid | 27 |
| Y | Total protein | 25 |
| Y | Albumin | 34 |
| Y | Total bilirubin | 29 |
| Y | Conj. bilirubin | 22 |
| Y | ALP | 35 |
| Y | GGT | 38 |
| Y | ALT | 38 |
| Y | AST | 38 |
| Y | LDH | 38 |
| Y | Amylase | 36 |
| Y | Lipase | 36 |
| **Cardiac:** | | |
| Y | CK | 40 |
| Y | CK-MB | 38 |
| ◔ | Troponin | 141 |
| **Diabetes / metabolic:** | | |
| Grey | Glucose - fast | 25 |
| Grey | Glucose - rand | 25 |
| P | HbA1c | 71 |
| Grey* | Lactate | 173 |
| **Lipids:** | | |
| Y | Triglycerride | 56 |
| Y | Total cholesterol | 38 |
| Y | HDL-cholesterol | 49 |
| **Anaemia:** | | |
| Y | HDL-cholesterol | 59 |
| Y | Transferrin | 83 |
| Y | Ferritin | 93 |
| Y | Vitamin B12 | 93 |
| P* | Folate - red cell | 131 |

| | | Rands |
|---|---|---|
| **Endocrinology:** | | |
| Y | TSH | 146 |
| Y | Free T4 | 123 |
| Y | Free T3 | 123 |
| Y | bHCG | 87 |
| Y | FSH | 93 |
| Y | LH | 93 |
| Y | Estradiol | 93 |
| Y | Progesterone | 96 |
| Y | Prolactin | 93 |
| Y | Testosterone | 93 |
| Y | SHBG | 93 |
| ◔ | PTH | 127 |
| Y | Cortisol | 93 |
| Y | Insulin | 93 |
| Y | AFP | 93 |
| **Cerebrospinal fluid:** | | |
| Grey | CSF glucose | 25 |
| | CSF chloride | 18 |
| | CSF protein | 22 |

### DRUGS

| | | |
|---|---|---|
| Y | Paracetamol | 80 |
| Y | Salicylate | 80 |
| Y | Lithium | 39 |
| Y | Methotrexate | 80 |
| Y | Tricyclics | 80 |
| Y | Phenytoin | 80 |
| Y | Phenobarbitone | 80 |
| Y | Epilim / Na valpr | 80 |
| Y | Carbamezepine | 80 |
| Y | Digoxin | 92 |
| Y | Theophylline | 80 |
| | Urine mandrax | 80 |

| | | trough | peak | |
|---|---|---|---|---|
| P | Cyclosporin | ☐ | ☐ | 154 |
| Y | Amikacin | ☐ | ☐ | 88 |
| Y | Gentamycin | ☐ | ☐ | 88 |
| Y | Vancomycin | ☐ | ☐ | 88 |

**Time of dose :**

### HAEMATOLOGY

| | | Rands |
|---|---|---|
| **General:** | | |
| P | FBC | 48 |
| P | Differential count | 26 |
| P | Film (morph) | 26 |
| P | Haemoglobim | 15 |
| P | White cell count | 15 |
| P | Platelet count | 18 |
| P | Reticulocytes | 24 |
| P | Malaria screen | 43 |
| ◔ | ESR | 24 |
| **Coagulation:** | | |
| B | INR | 39 |
| B | PTT | 44 |
| B | Fibrinogen | 28 |
| B | D-dimers | 216 |
| B | Thrombin time | 56 |
| B | Antithrombin III | 173 |
| B | Lupus anticoag | 197 |
| B | Protein C | 238 |
| B | Protein S | 293 |
| B | APC Resist | 205 |
| **Other:** | | |
| P | Coombs | 29 |
| © | CSF/Fld cytosp | 52 |

### VIROLOGY

| | | Rands |
|---|---|---|
| **HIV testing:** | | |
| Y | HIV serology | 97 |
| PP | HIV viral load | 318 |
| P | PCR : HIV | 387 |

**Hepatitis serology:**

Y Clinical hepatitis: 105 ea

☐ A ☐ B ☐ C

Y Hepatitis immunity:

☐ A ☐ B

| **Other serology:** | | IgM | IgG |
|---|---|---|---|
| | | 102 | 94 |
| Y | CMV | ☐ | ☐ |
| Y | EBV | ☐ | ☐ |
| Y | HSV | ☐ | ☐ |
| Y | VZV | ☐ | ☐ |
| Y | Rubella | ☐ | ☐ |
| ♦ | Measles | ☐ | ☐ |
| Y | Mumps | ☐ | ☐ |

| | | |
|---|---|---|
| **Viral isolation:** | | |
| P | CMV pp65 Ag | 323 |
| | Rapid RSV | 87 ea |
| | Rap. rota/adeno | 169 |
| | Culture (specify) | 87 |
| | PCR (specify) | var |

### IMMUNOLOGY

| | | |
|---|---|---|
| P | CD4 (PLG) | 64 |
| Y | CRP | 60 |
| Y | IgG, IgA, IgM | 55 ea |
| Y | Total compl | 97 |
| Y | Syphilis Serology | 16 |
| Y | ASOT / DNase B | 87 |
| Y | Toxoplasma | 94 |
| Y | ANF | 116 |
| Y | Anti ds-DNA | 116 |
| Y | Anti cardiolipin | 93 |
| Y | ANCA | 232 |
| Y | RF | 61 |
| Y | ENA | 115 ea |

### MICROBIOLOGY

**Specimen site:**

| | | |
|---|---|---|
| **General:** | | |
| Y | Routine MC&S | ≥81 |
| Y | CSF cell count, microscopy & culture | ≥141 |
| Y | Fungal M&C | ≥67 |
| Y | Blood culture | ≥101 |
| Y | Parasites | ≥57 |
| **TB investigation:** | | |
| | Microscopy | 32 |
| | Culture | ≥101 |
| | Sensitivity | ≥190 |
| **Antigen detection:** | | |
| | Cryptococcal Ag | 40 |
| | Pneumocystis | 87 |
| | Chlamydia | 116 |
| | C. difficile toxin | 217 |

**RECEIVED IN LAB**

### OTHER TESTS (ALL DISCIPLINES)

For full repertoire of tests, consult laboratory handbook

**# PROVINCIAL REQUIREMENTS :**

**Signature & PERSAL No. of Consultant:**

Specimens may be rejected if Patient name and number, Practitioner name, signature and Persal / Practice number, or Hospital / clinic / ward are omitted.

I hereby certify that I have considered the repertoire of tests requested as well as previous test requests for this patient and confirm that this form is duly completed and will not result in any fruitless and wasteful expenditure.

| SORT | LABEL |
|---|---|
| | |
