# Supplementary material for: The impact of physician education regarding the importance of providing complete clinical information on the request forms of thrombophilia-screen tests at Tygerberg hospital in South Africa
Source: PLoS One. 2020 Aug 6;15(8):e0235826. doi: 10.1371/journal.pone.0235826 (PMC7410402; doi:10.1371/journal.pone.0235826)
Supplement: S2 Appendix — (PDF) [file pone.0235826.s003.pdf]

Thrombophilia screen request form

Patient information:

Patient surname	First name
FN:	DOB:

Requesting Doctor Details:

Name:	Cell No:
Tel:	Signature:

Specimen info: specimen type?_______________________________________

Date taken: DD/MM/YYYY	Time taken:

Reason for screening/ Diagnosis: ____

Acute thrombosis: Yes ☐ No ☐

Number of thrombotic events

Episode	clot location	spontaneous or with other reasons	date of event
first			
2nd			
others			

First degree family history of thrombosis: Yes ☐ No ☐

Obstetric history:

Pregnant: Yes ☐ No ☐ If yes, how many weeks gestation?_____ weeks

History of pregnancy loss: Yes ☐ No ☐ Number of losses___ Date of last loss: DD/MM/YYYY

Medical history:

Acute attack of thrombosis: Yes ☐ No ☐ Current DIC: Yes ☐ No ☐

Liver failure: Yes ☐ No ☐ Renal failure Yes ☐ No ☐

Medication:

Anticoagulant:

Warfarin: Yes ☐ No ☐ If yes, when was last dose DD/MM/YYYY

Heparin: Yes ☐ No ☐ If yes, when was last dose DD/MM/YYYY

Oral contraceptive: Yes ☐ No ☐ Hormonal replacement therapy: Yes ☐ No ☐

Other Medications: Yes ☐ No ☐ If yes please specify ____________________________

Acquired	x	Tube	Hereditary	x	Tube	Genetic	x	Tube
LAC		Blue top	ATIII		Blue top	Prothrombin mutation		Purple top
			PS					
			PC			Factor V Leiden		
B2GP1		Yellow top						
Anticardiolipin								
